# Stearoyl-CoA Desaturase-1: Is It the Link between Sulfur Amino Acids and Lipid Metabolism?

**DOI:** 10.3390/biology4020383

**Published:** 2015-06-03

**Authors:** Soraia Poloni, Henk J. Blom, Ida V. D. Schwartz

**Affiliations:** 1 Post-Graduation Program in Genetics and Molecular Biology, Universidade Federal do Rio Grande do Sul, Av. Bento Gonçalves, 9500 Prédio 43323M, Porto Alegre, RS 91501-970, Brazil; E-Mail: spoloni@hcpa.ufrgs.br; 2 Laboratory of Clinical Biochemistry and Metabolism, Department of General Pediatrics, Adolescent Medicine and Neonatology, University Medical Centre Freiburg, Mathildenstrasse 1, Freiburg 79106, Germany; E-Mail: henk.blom@uniklinik-freiburg.de; 3 Medical Genetics Service, Hospital de Clínicas de Porto Alegre, Rua Ramiro Barcelos, 2350 Santa Cecília, Porto Alegre, RS 90035-903, Brazil

**Keywords:** lipoproteins, stearoyl CoA desaturase-1, SCD-1, homocysteine, cysteine, sulfur amino acids, homocystinuria

## Abstract

An association between sulfur amino acids (methionine, cysteine, homocysteine and taurine) and lipid metabolism has been described in several experimental and population-based studies. Changes in the metabolism of these amino acids influence serum lipoprotein concentrations, although the underlying mechanisms are still poorly understood. However, recent evidence has suggested that the enzyme stearoyl-CoA desaturase-1 (SCD-1) may be the link between these two metabolic pathways. SCD-1 is a key enzyme for the synthesis of monounsaturated fatty acids. Its main substrates C16:0 and C18:0 and products palmitoleic acid (C16:1) and oleic acid (C18:1) are the most abundant fatty acids in triglycerides, cholesterol esters and membrane phospholipids. A significant suppression of SCD-1 has been observed in several animal models with disrupted sulfur amino acid metabolism, and the activity of SCD-1 is also associated with the levels of these amino acids in humans. This enzyme also appears to be involved in the etiology of metabolic syndromes because its suppression results in decreased fat deposits (regardless of food intake), improved insulin sensitivity and higher basal energy expenditure. Interestingly, this anti-obesogenic phenotype has also been described in humans and animals with sulfur amino acid disorders, which is consistent with the hypothesis that SCD-1 activity is influenced by these amino acids, in particularly cysteine, which is a strong and independent predictor of SCD-1 activity and fat storage. In this narrative review, we discuss the evidence linking sulfur amino acids, SCD-1 and lipid metabolism.

## 1. Introduction

Methionine, homocysteine, cysteine and taurine metabolism are highly linked. These main sulfur amino acids are involved in several metabolic pathways such as glutathione synthesis, protein synthesis and the methylation of several substances, such as DNA, RNA, proteins and lipids [1,2,3]. Several reports have suggested that sulfur amino acids play a role in the regulation of lipid metabolism and body composition [4,5,6,7]. In this narrative review, we describe the possible connections between sulfur amino acids, SCD-1 and lipid metabolism.

**Figure 1:**
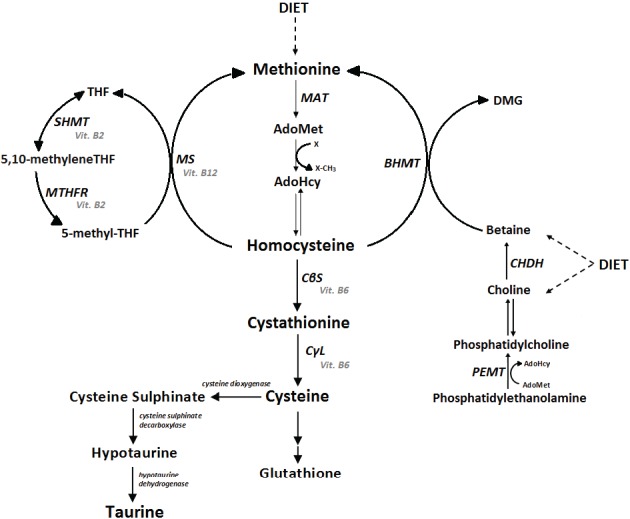
Metabolism of sulfur amino acids. MAT, methionine adenosyltransferase; AdoMet, *S*-adenosylmethionine; AdoHcy, *S*-adenosylhomocysteine; X, methyl acceptor; CβS, cystathionine β-synthase; CγL, cystathionine γ-lyase; MS, methionine synthase; THF, tetrahydrofolate; MTHFR, 5,10-methylene-THF reductase; SHMT, serine hydroxymethyltransferase; BHMT, betaine-homocysteine *S*-methyltransferase; DMG, dimethylglycine; CHDH, choline dehydrogenase; PEMT, phosphatidylethanolamine *N*-methyltransferase. Enzymes are shown in italics, and their cofactors are shown in gray.

The metabolism of sulfur amino acids is depicted in Figure 1. Methionine is an essential amino acid that is demethylated via two intermediate compounds, *S*-adenosylmethionine (AdoMet) and *S*-adenosylhomocysteine (AdoHcy). Homocysteine is a product of the transmethylation pathway, an amino acid that is not incorporated into proteins, and is considered toxic. Homocysteine may be metabolized by two different methods: through the transsulfuration pathway, where it is irreversibly degraded to cysteine; or through remethylation, where it is converted back to methionine.

Remethylation of homocysteine into methionine can occur through two alternative pathways. Homocysteine can be catalyzed by methionine synthase, which is a vitamin B_12_- and folate-dependent enzyme, or it can be catalyzed by betaine-homocysteine methyltransferase (BHMT), an enzyme present in liver and kidney that uses as methyl group donor betaine, which on its turn is formed by choline catabolism [3,8]. The transsulfuration pathway converts homocysteine into cystathionine and subsequently into cysteine. These reactions are catalyzed by two pyridoxal 5'-phosphate (PLP)-dependent enzymes.

Cysteine can be degraded through oxidative reactions that generate taurine or sulfate in a 2:1 ratio [2]. In addition, cysteine is used in the synthesis of proteins and glutathione, a powerful antioxidant. Taurine is the most abundant amino acid in animal tissues and used in the synthesis of bile salts, and it potentially acts as an antioxidant, membrane stabilizer and neurotransmitter [2,9].

The control of sulfur amino acid metabolism is a complex process that operates on several levels. AdoMet plays a central role in this regulation. When methionine levels increase, the concentration of AdoMet increases, favoring sulfur amino acid metabolism through the transsulfuration pathway, via activating cystathionine β-synthase (CβS) and inhibiting 5,10-methylene-tetrahydrofolate reductase (MTHFR). If methionine levels are low, such as during fasting, the reduced AdoMet levels do not activate CβS or inhibit MTHFR, thus resulting in the remethylation of homocysteine into methionine [3,8]. The enzyme activity is also influenced by factors such as protein intake, hormone levels, nutrient deficiencies, age and long-term changes in substrate levels [10].

Hyperhomocysteinemia is a condition characterized by elevated circulating levels of homocysteine (>15 μmol/L). Mild and moderate forms are frequent and present in 5%–10% of the population [11]. These forms are generally of multifactorial origin, and they are associated with higher mortality and incidence of several chronic diseases, such as stroke, dementia, Alzheimer’s disease, bone fractures and heart failure [12]. Causes of mild/moderate hyperhomocysteinemia are described in Table 1.

The severe form of hyperhomocysteinemia (homocysteine >50 μmol/L) is rare, usually monogenic (autosomal recessive inheritance), and is caused by pathogenic variations in genes involved in homocysteine clearance, such as *C*β*S*, *MTHFR* and *methylmalonic aciduria and homocystinuria type C* (*MMACHC*). CβS deficiency is the most common type of severe hyperhomocysteinemia, with an estimated worldwide prevalence of 1 in 344,000 individuals [13]. In humans, this deficiency results in classical homocystinuria, a disease characterized by increased plasma levels of homocysteine, methionine and AdoMet and decreased levels of cystathionine and cysteine. The classical clinical manifestations are lens dislocation, thromboembolism, osteoporosis, cognitive deficit and psychiatric disorders [13,14]. The so-called Marfanoid phenotype, which includes increased height, dolichostenomelia and lean biotype, is also common [13,15].

**Table 1:** Summary of the main causes of mild and moderate hyperhomocysteinemia.

Etiology	Characteristics
**Vitamin B12 deficiency**	Vitamin B12 is used in homocysteine remethylation. The groups at higher risk for this deficiency are vegetarians and the elderly, with the deficiency rates reaching 20% for the latter. Deficient absorption is likely the main etiological factor in elderly individuals because intrinsic-factor deficiency, which is fundamental for the absorption of B12, is common in this age group [62].
**Folate deficiency**	Folate is also involved in the remethylation of homocysteine, and its deficiency is more common in individuals with increased needs, such as pregnant women and alcoholics. In addition, a frequent polymorphism in the *MTHFR* gene (c.677C > T) makes the enzyme thermolabile and decreases enzymatic activity in homozygotes to 70%. If their folate status is low to normal, homocysteine can increase dramatically [63].
**Medicines**	The chronic use of certain drugs, such as methotrexate, theophylline, phenytoin, carbamazepine and valproic acid, is known to increase homocysteine levels [64,65].
**Other causes**	Additional causes include chronic renal disease, cancer, hypothyroidism, liver failure, smoking, alcohol and coffee intake, age and hormonal disorders [64,65].

MTHFR: 5,10 methylenetetrahydrofolate acid reductase.

Severe MTHFR deficiency is characterized by hyperhomocysteinemia, homocystinuria and low-to-normal levels of methionine. This form of homocystinuria is rare with approximately 100 cases described [16]. The main clinical manifestations include seizures, cognitive impairments, gait disturbances and isolated thromboembolic episodes. In addition to the mutations that cause severe deficiency, polymorphisms in the *MTHFR* gene are highly prevalent in the population. The most prevalent are the c.677C > T and c.1298A > C. The first is associated with mild hyperhomocysteinemia, especially when associated with low folic acid status [14].

Another cause of severe hyperhomocysteinemia (over 500 cases described) [17] is the *cbl*C type of homocystinuria with methylmalonic aciduria, which is caused by pathogenic variations in the *MMACHC* gene, which codes for a protein involved in the binding and intracellular trafficking of cobalamin. The most frequent symptoms are developmental delays, psychiatric disorders, microangiopathy, and ocular and hematologic abnormalities [13,14].

## 2. Sulfur Amino Acids and Lipid Metabolism

Several studies have indicated the role of sulfur amino acids in the regulation of lipoprotein metabolism. In wild-type mice, methionine supplementation promotes hypercholesterolemia [18]. In turn, taurine is inversely related to very-low-density lipoprotein (VLDL), low-density lipoprotein (LDL)-cholesterol and high-density lipoprotein (HDL)-cholesterol in humans and rodents, and its supplementation has hypocholesterolemic action in rats [4,5]. In population-based studies, plasma cysteine levels were found to be positively associated with levels of several lipoproteins, such as total cholesterol, LDL, apolipoprotein B (ApoB) and triglycerides [6,7].

Regarding homocysteine, an inverse association between this amino acid and lipoproteins, especially HDL, has been well described in humans [19,20,21] and various animal models of hyperhomocysteinemia. CβS homozygous deficient mice (CβS^−/−^) present plasma homocysteine levels that are 40-fold higher than that of the wild type, which is comparable to the levels observed in patients with classical homocystinuria. They also present low weight gain and have low survival rates [22]. In this mouse model, increased hepatic levels of triglycerides and non-esterified fatty acids, low levels of ApoB100 and ApoA-I and reduced serum concentrations of total cholesterol and HDL were observed [23,24].

Mice with MTHFR deficiency have excess homocysteine but normal levels of cysteine. They also exhibit lipid metabolism changes, including reduced levels of ApoA-I and an inverse association of homocysteine with ApoA-I and HDL [25]. The clinical features include developmental retardation with cerebellar pathology, growth failure and abnormal aortic lipid deposition [26]. Despite consistent evidence of lipid metabolism modulation by these sulfur amino acids, their mechanism of action is not well understood. Recently, several studies have suggested that sulfur amino acids modulate the expression of stearoyl-CoA desaturase-1 (SCD-1), a key enzyme in the hepatic synthesis of monounsaturated fatty acids [27,28]. The putative mechanisms by which sulfur amino acids might regulate lipid metabolism and SCD-1 expression are summarized in Table 2.

**Table 2:** Proposed mechanisms by which sulfur amino acids influence lipid metabolism and may potentially regulate SCD-1 expression.

Amino Acid	Effect on Lipid Metabolism	Effect on SCD-1 Regulators
**Methionine**	‒ Induction of ApoA1 synthesis in rats [66]	‒ Unknown
**Homocysteine**	‒ Suppression of ApoAI synthesis in mice [27] ‒ Regulation of ApoB100 levels in mice [24] ‒ Induction of *CYP7A1* expression in rats [66]	‒ Induction of leptin secretion in humans [68,69] ‒ Activation of the transcription factors SREBP-1c and PPARα in humans and mice [70,71]
**Cysteine**	‒ Regulation of ApoE and ApoA1 levels in mice [25,67]	‒ Modulation of PPARγ, SREBP-1c and estrogen receptor-α expression in humans and animals [72–75]
**Taurine**	‒ Reduction in ApoB and VLDL secretion in human liver cells [4] ‒ Modulation of gene expression (*CYP7A1*, *LXR*) in animal models [66] ‒ Increased excretion of fecal bile acid in several animal models [4]	‒ Modulation of LXR-α (direct ligand) and *SREBP-1* expression in macrophages [76] ‒ Regulation of insulin and leptin secretion in animal models [5,77]

SCD-1, stearoyl-CoA desaturase-1; ApoA1, Apolipoprotein A1; ApoB100, Apolipoprotein B100; CYP7A1, cytochrome P450, family 7, subfamily A, polypeptide 1; ApoE, apolipoprotein E; LXR, liver X receptor; SREBP-1c, sterol regulatory element binding transcription factor 1; PPARα, peroxisome proliferator-activated receptor alpha; PPARγ, peroxisome proliferator-activated receptor gamma.

## 3. SCD-1: Localization, Function and Regulation

The enzyme SCD-1 is bound to the endoplasmic reticulum and responsible for the synthesis of monounsaturated fatty acids. Together with NAPH, cytochrome b5 reductase and cytochrome b5, SCD-1 introduces a single double bond at the Δ9,10 of long-chain acyl-CoA substrates, and its main substrates are palmitic (C16:0) and stearic (C18:0) acids, although other substrates containing 9–20 carbons can also bind to the enzyme [23,24]. The products palmitoleic acid (C16:1) and oleic acid (C18:1) are the main fatty acids present in triglycerides, cholesterol esters and membrane phospholipids [28,29,30].

In humans and rodents, SCD-1 is mainly expressed in liver and adipose tissue (brown and white); however, expression at lower levels is also observed in the kidneys, spleen, heart and lungs [27]. The half-life of the enzyme is only 3–5 h, and its degradation occurs in the microsomes [31]. Its gene contains several binding sites for transcription factors that regulate its expression. Among the main factors is SREBP-1c (sterol regulatory element binding protein), a transcription factor that regulates the biosynthesis of fatty acids, LXRα and LXRβ receptors (which activate genes associated with cholesterol transport), PPARs (peroxisome proliferator-activated receptors), nuclear receptors involved in adipocyte differentiation and lipid storage and estrogen receptors, which also regulate lipogenesis [28,32].

Expression of the *SCD-1* gene is regulated by several intrinsic and extrinsic factors, including leptin, a hormone involved in appetite regulation and energy expenditure. It is believed that most of leptin’s actions in the liver occur through SCD-1 suppression, and that this effect is independent of insulin and SREBP-1c [32,33]. Diet is also an important modulator of SCD-1 expression, with high consumption of polyunsaturated fatty acids suppressing the enzyme and consumption of saturated fatty acids, cholesterol and carbohydrates increasing its expression. Alcohol consumption and smoking appear to upregulate SCD1 activity, whereas physical activity has the opposite effect [28,34,35,36,37].

The effects of SCD-1 deficiency have been reported in animal models. *SCD-1* global knockout mice (SCD-1 −/−) exhibit reduced levels of cholesterol esters and triglycerides, and dietary supplementation with monounsaturated fatty acids is not capable of normalizing the production of these compounds [30]. SCD-1 −/− mice also exhibit lower fat accumulation in adipose tissue regardless of higher energy consumption as well as higher basal energy expenditure and improved insulin sensitivity. In addition, these animals are resistant to weight gain and diet-induced fat accumulation [38].

SCD-1’s effect on the phenotype, however, appears to be related to SCD-1 expression in specific tissues. Mice that only present SCD-1 deficiency in the liver have a similar phenotype to that of the wild type fed a chow diet. However, on a high-carbohydrate diet, SCD-1 −/− mice showed less weight gain and a reduction in plasma triglycerides compared with the controls, and they were also protected from diet-induced liver steatosis. The same changes did not occur when the mice were fed a high-fat diet, and they presented similar gain weight gain as their littermate controls and developed liver steatosis. The level of insulin sensitivity did not differ between treatments [39]. In addition, the food intake, insulin sensitivity and fat mass in mice with the adipose SCD-1 deletion were similar to that of the controls, and they were not protected from diet-induced obesity [40]. In contrast, mice with skin-specific deletions of SCD-1 showed significantly increased energy expenditures and were protected from high-fat diet-induced obesity [41].

Increased β-oxidation is the main proposed mechanism through which SCD-1 deficiency results in this anti-obesogenic phenotype. Because SCD-1 deficiency has negative effects on the synthesis of VLDL and triglycerides, an increase in the pool of saturated acyl CoAs would result and allosterically inhibit acetyl CoA carboxylase. This enzyme converts acetyl CoA into malonyl CoA. The decrease in malonyl CoA levels counteracts the inhibition of carnitine palmitoyltransferase 1 (CPT-1), thus allowing the entry of fatty acids into the mitochondria for oxidation [32].

In humans, product/precursor ratios in either plasma or serum have been used to estimate SCD-1 activity. Population-based studies have found strong positive associations between the indices 16:1n-7/16:0 and 18:1n-9/18:0 and obesity markers such as the body mass index (BMI), waist circumference and body fat mass as evaluated by dual-energy X-ray absorptiometry [35,36,42]. Other cardiovascular risk factors are also associated with SCD-1 indices. In a study with 134 healthy men, plasma levels of palmitoleic acid, one of the main products of SCD-1, were strongly and independently associated with triglyceridemia [43]. In another study, high SCD-1 activity (estimated through the indices mentioned above) was found to be a predictor of the development of metabolic syndrome in a cohort of 1558 middle-aged men [44]. A similar association was observed for the development of hyperglycemia [45]. The development of other diseases, such as cancer, bone fractures and hepatic steatosis, has also been associated with SCD-1 expression [28].

## 4. Evidence that Sulfur Amino Acids Influence SCD-1 Expression

Knowledge of the effects of sulfur amino acids on the regulation of SCD-1 expression has increased in recent years, especially because of studies using animal models. A recent review [46] of animal model knockouts for several enzymes (CβS, glutamate cysteine ligase modifier subunit, cystathionase, BHMT, PEMT, gamma glutamyltransferase and cysteine dioxygenase) involved in sulfur amino acid metabolism showed that alterations in these pathways, especially when related to low cysteine and choline levels, resulted in similar anti-obesogenic phenotypes in rodents. However, because of the complexity of this regulation and its interdependence with other metabolic pathways, the individual role of each amino acid remains unclear.

Low weight gain and a significant decrease in body fat mass (46% in females and 62% in males) were observed in a study using TgI278T CβS^−/−^ mice, an animal model of classical homocystinuria that has a mutant human *C*β*S* gene containing the common p.I278T pathogenic variation. This mutation was found in 16% of the homocystinuria alleles studied in the world, presents a pan-ethnic distribution and is associated with pyridoxine responsiveness (http://cbs.lf1.cuni.cz/index.php). A liver microarray analysis revealed SCD-1 to be the transcript with the largest magnitude change in these mice, with a seven-fold decrease in gene expression relative to the control. The hepatic levels of the enzyme were also reduced by 54% for group TgI278T CβS^−/−^, but no changes were observed in visceral and subcutaneous fat [47].

The effects of sulfur amino acids on body composition and lipid metabolism have also been evaluated in mice subjected to a methionine-restricted (MR) diet. These animals exhibited reduced levels of methionine (62%), taurine (64%), cysteine (44%) and cystathionine (44%) and a 2.5-fold increase in homocysteine concentration [48]; and they displayed lower weight gains despite their higher energy consumption. The effect of treatment with cysteine (MR + Cys) was tested on this animal model. Following 12 weeks of treatment, the MR + Cys group exhibited weight gain and adiposity similar to that of the control group. Certain lipoprotein changes observed in the MR group (low levels of triglycerides and higher levels of LDL) were reverted by the cysteine treatment. In addition, the reduced levels of leptin, insulin growth factor (IGF)-1 and insulin and elevated levels of adiponectin observed for the MR group were normalized following treatment with cysteine. The decreases in the SCD1-16 and SCD1-18 indices and low hepatic expression of SCD-1 observed in the MR group were also reverted in the MR + Cys group. Supplementation with cysteine completely normalized the plasma levels of cysteine and cystathionine but only partially corrected the taurine and homocysteine concentrations [49].

The role of taurine was also tested in this animal model. The remaining sulfur amino acid concentration, weight gain, body composition, lipid profile, hepatic activity and SCD-1 expression were all unaffected by supplementation with taurine (MR + Tau). In fact, the taurine treatment presented further decreases of body fat, suggesting that this amino acid is not a direct mediator of adipogenesis [50]. In another study, mice lacking the glutamate-cysteine ligase modifier subunit gene (Gclm(−/−)) were studied. These animals present glutathione deficiency and low cysteine plasma levels of cysteine [51]. The Gclm(−/−) mice presented lower body weight (primarily because of lower fat mass), improved glucose tolerance and higher basal metabolic rates compared with that of the wild type, and a high-fat diet did not induce significant changes in these phenotypes. The SCD-1 expression was markedly downregulated in the liver at only 17% of the wild type [52].

Changes in choline/betaine metabolism, which is highly interconnected with sulfur amino acid metabolism, have also been associated with changes in energy, lipid and glucose metabolism [53]. Deficiency of the enzyme BHMT (Figure 1), which remethylates homocysteine to methionine, results in increased levels of betaine and homocysteine and decreased levels of choline and cysteine in mice [54]. BHMT-deficient mice exhibit lower body-fat mass, reduced triglyceride synthesis, improved sensitivity to insulin and higher energy expenditure. These changes are not associated with changes in food intake, lipid absorption, lipolysis or thermogenesis [55]. Dietary deprivation of methionine and choline in mice also results in decreased body-fat mass; higher energy expenditures; lower serum triglyceride, leptin, insulin and glucose concentrations; and suppressed hepatic SCD-1 expression. In this animal model, downregulation of SCD-1 was associated with hepatic steatosis [56].

Studies of the relationship between sulfur amino acids and SCD-1 activity in humans are scarce. In the Hordaland Health Studies (HUSK) and Hoorn European cohort studies, a positive and independent association was observed between plasma cysteine levels and the SCD-16 index. The plasma levels of total cholesterol and triglycerides were also positively associated with the SCD-16 index. No consistent associations were found between the SCD-16 index and homocysteine, methionine, AdoMet, cystathionine or glutathione plasma concentrations [57]. These findings are consistent with other epidemiological studies indicating that cysteine is an independent predictor of obesity [58,59,60]. In addition, patients with classical homocystinuria who also have low cysteine concentrations have reduced body mass and exhibit lipid metabolism abnormalities [21,61]. A positive association between BMI and cysteine levels has also been observed in these individuals [61]. This phenotype has not been described in other forms of homocystinuria in humans who have normal cysteine levels.

## 5. Conclusions

The regulation of lipid metabolism by sulfur amino acids has gained more attention in recent years. Although the mechanisms are far from fully understood, animal and population-based studies have suggested that one or more sulfur amino acids affect lipoprotein production and lipid storage in adipose tissues and indicated that this action is possibly mediated by SCD-1. Although the most consistent evidence indicates cysteine to be the main modulator, other sulfur amino acids or choline and its derivatives may produce independent or additional effects on lipid metabolism.

Because SCD-1 appears to be strongly involved in the etiology of metabolic syndrome, understanding the regulation of this enzyme may assist in the development of new therapies and prevention strategies for several chronic diseases, such as obesity, diabetes, dyslipidemia and arteriosclerosis. Changes in sulfur amino acid metabolism, especially hyperhomocysteinemia, are frequent in the human population, further reinforcing the importance of these findings.
